# Transcription coupled repair and biased insertion of human retrotransposon L1 in transcribed genes

**DOI:** 10.1186/s13100-017-0100-5

**Published:** 2017-12-06

**Authors:** Geraldine Servant, Vincent A. Streva, Prescott L. Deininger

**Affiliations:** 1 Tulane University, Tulane Cancer Center and the Department of Epidemiology, 1430 Tulane Ave, New Orleans, LA 70112 USA; 2 Present Address: Division of Infectious Diseases, Boston Children’s Hospital and Harvard Medical School, 300 Longwood Ave, Boston, MA 02115 USA; 3 Tulane Cancer Center, SL66, Tulane University Health Sciences Center, 1430 Tulane Ave., New Orleans, LA 70112 USA

**Keywords:** L1 retrotransposon, Transcription-coupled repair, Target-primed reverse transcription, DNA repair, Mutagenesis

## Abstract

**Background:**

L1 retrotransposons inserted within genes in the human genome show a strong bias against sense orientation with respect to the gene. One suggested explanation for this observation was the possibility that L1 inserted randomly, but that there was negative selection against sense-oriented insertions. However, multiple studies have now found that *de novo* and polymorphic L1 insertions, which have little opportunity for selection to act, also show the same bias.

**Results:**

Here we show that the transcription-coupled sub-pathway of nucleotide excision repair does not affect the overall rate of insertion of L1 elements, which is in contrast with the regulation by the global sub-pathway of nucleotide excision repair. The transcription-coupled subpathway does cause a strong bias against insertion in the sense orientation relative to genes.

**Conclusions:**

This suggests that a major portion of the L1 orientation bias might be generated during the process of insertion through the action of transcription-coupled nucleotide excision repair.

**Electronic supplementary material:**

The online version of this article (10.1186/s13100-017-0100-5) contains supplementary material, which is available to authorized users.

## Background

Sequencing of the human genome revealed that transposable elements make up almost half of the genome [1, 2]. The long interspersed element L1 is the only active, autonomous retrotransposon in the human cells and constitutes 17% of the genome. L1 inserts are relatively randomly distributed in genic and intergenic regions, with the elements showing a genomic preference for AT-rich regions [3]. However, L1 copies within genes show a significant enrichment for the antisense orientation. It has been proposed that this orientation bias may be caused by a selection process limiting transcriptional interference with gene expression [3]. However, a similar trend is observed with published *de novo* inserts recovered in HeLa cells using an engineered L1 element, although there are insufficient data for that to reach significance [4–6], and somatic L1 insertions identified in brain cells [7]. These latter findings would be expected to be subjected to much less selection and raise the possibility for an insertion-related mechanism controlling L1 insertion in actively transcribed genes, in a gene-orientated manner.

**Fig. 1:**
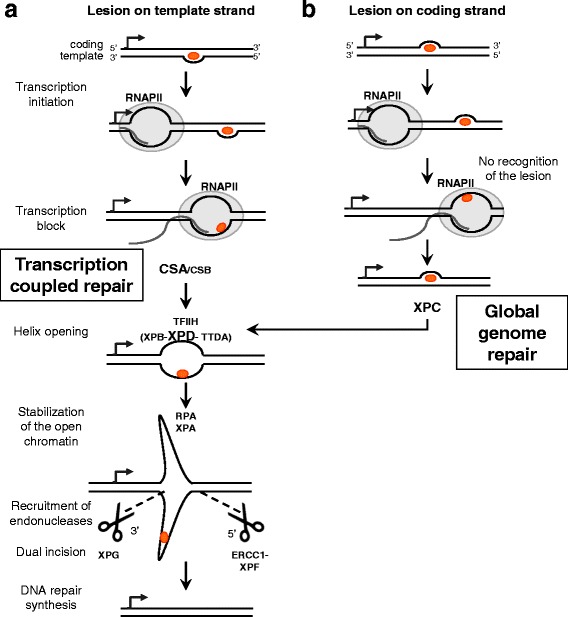
The predicted influence of NER sub-pathways on the coding or template damaged strand of an actively transcribed gene. **a** and **b** Schematic representation of the repair of a bulky lesion located on the template strand (panel **a**) or on the coding strand (panel **b**) of an active gene. If the lesion is on the template strand (panel **a**), read by RNAPII during the transcription process, the lesion causes the RNAPII complex to stall. CSA and CSB proteins are the sensors of stalled RNAPII and recruit the transcription complex TFIIH to the site of the lesion. The helicase activities, XPB and XPD, of the TFIIH complex open the chromatin around the lesion. XPA and RPA stabilized the open structure of the chromatin. The endonucleases, ERCC1-XPF in 5′ and XPG in 3′ cleave the damaged strand. The gap is then filled by DNA repair polymerases and ligases. If the lesion is on the coding strand (panel **b**) and therefore not read by the RNAPII complex, the lesion does not interfere with the enzyme processivity and the gene is transcribed. The lesion can be later recognized by the XPC complex, the lesion binding proteins in the global genome repair (GGR), the second NER sub-pathway. After the lesion recognition step, both GGR and TCR are identical. In bold are the factors controlled for their impact on L1 insertion regulation in the present study

Transcription-coupled repair (TCR), a sub-pathway of nucleotide excision repair (NER), is a DNA repair pathway that excises helix distorting lesions. These lesions are typically caused by UV-light exposure or chemical compounds and they block the RNA polymerase II (RNAPII) processivity on the template strand of transcribing genes ([8] and Fig. 1). CSA and CSB (Cockayne Syndrome proteins A and B), the sensor proteins of the pathway, are recruited to the stalled RNAPII complex and initiate the excision process of the damaged strand. If the bulky DNA lesion is located on the coding strand of the gene or in an untranscribed genomic region, they do not interfere with the transcription process and are then subject to the slower, global genome repair (GGR) NER sub-pathway ([9, 10] and Fig. 1). After lesion recognition, the TCR and GGR mechanisms converge on a common series of steps. Briefly, the DNA helix is opened by the helicase proteins, XPD and XPB, of the TFIIH complex [11]. The open DNA structure is then stabilized by XPA-RPA proteins [12, 13]. ERCC1-XPF and XPG endonucleases cleave the damaged strand at 5′ and 3′ ends of the lesion [14, 15].

We have recently shown that several proteins of the NER pathway, notably two central proteins of the DNA repair, XPD and XPA [16], as well as the endonuclease ERCC1-XPF [17] and the lesion binding protein XPC of the GGR pathway, limits L1 retrotransposition [16]. In cells with mutations in these genes, the L1 retrotransposition rate increased and generated larger tandem site duplications(TSDs) at the insertion site that are abnormally large [4–6, 16, 18]. As the GGR pathway can inhibit L1 insertions, we hypothesize that the TCR sub-pathway may also serve the same role. The TCR sub-pathway is only active on the portion of the genome that is actively transcribing in any given cell ([8] and see Discussion) while the GGR sub-pathway activity would continue to protect the majority of the genome. Because of this, we expect that TCR would not greatly affect the overall rate of retrotransposition. However, we hypothesize that it might generate a strong bias against L1 insertions in the template strand of transcribed genes, thereby helping to explain the observed bias in orientation of L1 elements within genes.

## Methods

### Cell lines and culture conditions

HeLa cells (ATCC CCL2) were grown in eMEM supplemented with 10% Fetal Bovine Serum, 0.1 mM non-essential amino acids (Life Technologies) and 1 mM sodium pyruvate (Life Technologies) at 37° in a 5% carbon dioxide environment. The following cell lines were obtained from the Coriell Cell Repository: CSA-SV40 transformed fibroblasts (GM16094), XPC-SV40 transformed fibroblasts (GM15983), XPD- SV40 transformed fibroblasst (GM08207), the stably complemented version of XPD- cell line (XPD+) (GM15877). XPC-, XPD- and CSA- cell lines were grown in eMEM supplemented with 10% Fetal Bovine Serum, 0.1 mM non-essential amino acids (Life Technologies) at 37° in a 5% carbon dioxide environment. XPD+ cell line was grown in the DMEM supplemented with 10% Fetal Bovine Serum (Life Technologies). A stably complemented version of the CSA- cell line (CSA+) was generated in this study by transfecting CSA- cells with a CSA cDNA expression vector (# EX-S0507-M67, GeneCopoeia) along with a hygromycin selection vector to allow selection for integration. CSA+ cells are maintained in eMEM medium supplemented with 10% Fetal Bovine Serum (Life Technologies), 0.1 mM non-essential amino acids (Life Technologies) and 200 μg/mL hygromycin at 37° in a 5% carbon dioxide environment.

### Plasmids

JM102/L1.3 contains the CMV promoter upstream of the L1.3 element deleted for the 5′ UTR and the *mneo* indicator cassette cloned in pCEP4 plasmid [19].

JM102/D702A/L1.3 derives from JM102/L1.3 and contains the reverse transcriptase deficient mutant of an L1.3 element and the *mneo* retrotransposition cassette cloned in pCEP4 vector [19].

TAM102/L1.3 contains the CMV promoter upstream of the L1.3 element deleted for the 5′ UTR and the *mblastI* indicator cassette cloned in pCEP4 vector [20].

TAM102/D702A/L1.3 derives from TAM/L1.3 and contains the reverse transcriptase deficient mutant of an L1.3 element and the *mblastI* indicator cassette cloned in pCEP4 vector [20].

TAM102/H230A/L1.3 derives from TAM102/L1.3 and contains the endonuclease deficient mutant of the L1.3 element and the *mblastI* indicator cassette cloned in pCEP4 vector [20].

# EX-S0507-M67 (GeneCopoeia) contains the CSA cDNA driven by CMV promoter and a hygromycin resistance gene in pReceiverM67 vector.

The synL1_neo vector used for the recovery of *de novo* L1 inserts was previously described [21].

The pIRES2-EGFP vector (Clontech) contains a neomycin resistance gene expressed from a SV40 promoter. The vector contains a multi-cloning site upstream of an IRES and eGFP marker. The cloned gene and eGFP marker are expressed from the CMV promoter on the same transcript.

All plasmid DNA were purified by Maxiprep kit (Qiagen). DNA quality was also evaluated by the visual assessment of ethidium bromide stained agarose gel electrophoresed aliquots.

### Retrotransposition assays

**Fig. 2:**
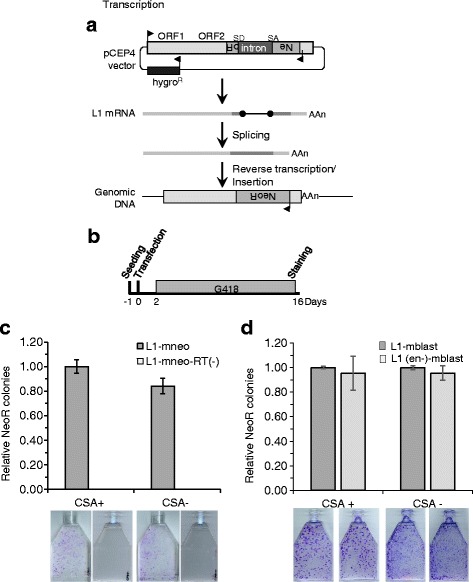
L1 retrotransposition rate is not significantly different in CSA-deficient cells (CSA-) and in the stably complemented CSA-deficient cells (CSA+). **a** Schematic of L1 retrotransposition assay. The L1.3 element tagged at the 3′ end with the *mneo* retrotransposition sensor is inserted in a pCEP4 vector (JM102/L1.3 vector). The retrotransposition cassette consists of a neomycin resistance (NeoR) gene in antisense orientation relative to the L1 element and expressed from its own promoter. The NeoR gene is not functional in the retrotransposition cassette because it is interrupted by an intron in L1 sense orientation. The NeoR gene becomes functional only after transcription, splicing, reverse transcription of L1 mRNA and insertion of L1 cDNA. **b** Schematic of the timing of the L1 retrotransposition assay. CSA- and CSA+ cells are seeded the day before transfection with JM102/L1.3 (L1.3-mneo-WT) or JM102/D702A/L1.3 (L1.3-mneo-RT(−)) expression vector. Two days after transfection, G418 selection is added to the growth medium and cells are kept under selection for 14 days. At the end of the assay, cells are fixed and stained and the number of NeoR colonies is determined. **c** CSA-deficient cells (CSA-) and the stably complemented version (CSA+) were transfected with JM102/L1.3 (L1-mneo) or JM102/D702A/L1.3 (L1-mneo-RT(−)) construct. Colony formation was assayed after two weeks under neomycin selection. The graph shows the relative colony number (average ± S.D.) of three independent experiments. Values are normalized to L1.3 WT vector. No significant differences (*p* > 0.05, two-tailed two sample Student’s T-test) were observed between the L1-mneo expression constructs in the different CSA+ and CSA- cells. Representative examples of NeoR colony formation from L1 retrotransposition assay in CSA+ and CSA- cells were presented below the graph. No colonies were detected with the L1 element with a defective RT. **d** CSA-deficient cells (CSA-) and the complemented version (CSA+) were co-transfected with TAM102/L1.3 (L1 mblast), or TAM102/H230A/L1.3 (L1 (en-)-mblast) construct and pIRES2-EGFP vector, a vector carrying a constitutive NeoR expression cassette. Colony formation due to random integration of this transfected plasmid was assayed after two weeks under neomycin selection. The L1 expression constructs were only included as a functional L1 and a defective (en-) L1 so that the experiment can simultaneously test for differences in the CSA- and CSA+ cells for transfection, colony formation and potential toxicity from the L1. The graph shows the relative colony number (average ± S.D.) of three independent experiments. Values are normalized to L1.3 WT vector. No significant differences (*p* > 0.05, two-tailed two sample Student’s T-test) were observed between the different L1 expression constructs in the different cell lines. Representative examples of NeoR colony formation from this L1 toxicity assay in CSA+ and CSA- cells are presented below the graph

Briefly, 5 × 10^6^ CSA+ and CSA- cells were seeded in T75 flasks. Cells were transfected the next day at about 90% confluence using Lipofectamine 2000 (Life Technologies) following the manufacturer’s protocol. Cells were transfected with 3 μg of L1.3 or L1.3-RT (−) construct tagged with the *mneo* retrotransposition cassette (JM102/L1.3 or JM102/D702A/L1.3) in T75 flasks. Two days after transfection, cells were selected for the transposition events in medium, containing 500 μg/mL Geneticin (Life Technologies). After 14 days, cells were fixed and stained with crystal violet solution (0.2% crystal violet in 5% acetic acid and 2.5% isopropanol) (Fig. 2b). Each assay was performed in triplicate. The number of neo^R^ colonies was counted in each flask.

### L1 toxicity and colony formation assay

L1 toxicity and colony formation assays were performed using the L1 episomal and the pIRES2-EGFP vectors. Briefly, 5 × 10^6^ CSA+ and CSA- cells were seeded in T75 flasks. Cells were transfected the next day at about 90% confluence using Lipofectamine 2000 (Life Technologies) following the manufacturer’s protocol. Cells were transfected with 3 μg of L1.3, or L1.3-EN (−) construct tagged with the *mblast* retrotransposition cassette (TAM102/L1.3, or TAM102/H230A/L1.3) and 0.5 μg of pIRES2-EGFP vectors (pIRES2-GFP was used because it contains a G418 resistance cassette). Cells were selected for the presence of the pIRES2-EGFP plasmid in selective medium containing 500 μg/ml geneticin (Life Technologies) for 14 days. The cells were then fixed and stained with crystal violet solution (0.2% crystal violet in 5% acetic acid and 2.5% isopropanol). The number of neo^R^ colonies was counted in each flask.

### RT-qPCR

Total RNA were extracted from a confluent T75 flask, using TRIzol Reagent (Life Technologies). We then carried out chloroform extraction and isopropanol precipitation. RNA was suspended in 100 μL of DEPC-treated water. The cDNA was synthetized using the Reverse Transcription System (Promega), following the manufacturer’s protocol. Briefly 1 μg of total RNA was denatured at 65° for 5 min. The reverse transcription reaction was primed with Oligo(dT)_15_ primers and incubated at 42° for 1 h in a thermocycler (BioRad, C1000 Touch). The enzyme was then heat-inactivated at 85° for 5 min. The PCR amplification of CSA cDNA was performed using previously published primers [22]. Meanwhile, the PCR amplification of beta-actin cDNA was performed as a control of the assay. The PCR products were analyzed on a 1% agarose gel and the bands were gel extracted and cloned into TOPO-TA (Life Technologies). Cloned PCR products were Sanger sequenced using M13 forward and reverse primers. Samples were sent for Sanger sequencing to Elim Biopharmaceuticals, Inc., Hayward, California. Lasergene 10 SeqBuilder software was utilized for sequence analysis and the sequences were compared to the reference cDNA using BLAST software (website: https://blast.ncbi.nlm.nih.gov/Blast.cgi).

### Recovery of *de novo* L1 insertions

*De novo* L1 insertion recovery was performed as previously described [16]. Briefly, 5 × 10^6^ CSA- and CSA+ cells were transfected with 3 μg synL1_neo rescue vector [21] using Lipofectamine 2000 reagent (Life Technologies). Cells were selected with 500 μg/mL of Geneticin (Life Technologies) for 14 days to allow for colony formation. Neo^R^ cells were harvested by trypsinization and genomic DNA was extracted using a Qiagen DNeasy Blood and Tissue kit. Genomic DNA was digested with 100 U of *Hin*dIII (NEB) overnight at 37°. The following day, digested genomic DNA was self-ligated using 1200 U T4 DNA ligase (NEB) in a volume of 1 mL overnight at room temperature. DNA was purified and concentrated using centrifugal filters (Amicon Ultra, 0.5 mL, 50 K, Millipore). Purified DNA was transformed by electroporation into competent DH5α *E. coli* (Life Technologies). Individual kanamycin-resistant colonies were grown and plasmid DNA was harvested using SV Wizard miniprep kit (Promega). The 5′ end of the *de novo* L1 insertion was sequenced using primers specific to the L1 rescue plasmid and primer walking until the 5′ end of the insert was recovered as described in [20]. Because sequencing through a long adenosine tract at the 3′ end of the L1 insertions is not effective, the 3′ flanking genomic region was sequenced by ligation mediated PCR based on [23, 24]. Briefly, a pool of five to six L1 rescue vectors was digested with *Stu*I (NEB) to relax supercoils, and then sheared by sonication using a Bioruptor (Diagenode, high, 30 s on, 90 s off, for 12 min). Sheared plasmid DNA was primer extended using an oligo specific to the 3′ end of the synL1_neo rescue plasmid (3′_rescue_1: 5′ ATATATGAGTAACCTGAGGC 3′ or 3′_rescue_1_secondpA: 5′ GTGGGCATTCTGTCTTGTTC 3′). Duplexed T-linkers were ligated using 10 U T4 DNA ligase and PCR was performed using the primers: linker specific (5′ ACACTCTTTCCCTACACGACGCTCTTCCGATCT 3′) and 3′_rescue_1 (or 3′_rescue_1_secondpA) primer. PCR was carried out with these steps: initial denaturation at 94°, 20 cycles of 94° for 30s, 60° for 1 min, 72° for 1 min, and a final extension for 10 min at 72°. PCR reactions were run on a 1% agarose gel and a light smear between 400 and 700 nt was gel extracted with the Qiaquick gel extraction kit (Qiagen). One μL of gel extracted DNA was subject to an additional 15 cycles of PCR amplification as described above using linker specific and nested 3′ rescue vector primers (3′_rescue_2: 5′ TGAGTAACCTGAGGCTATGCTG 3′ or 3′_rescue_2_secondpA: 5′ TTCTGTCTTGTTCCGGTTCTTAAT 3′). The nested PCR product was run on a 1% agarose gel and the resulting smear was gel extracted and cloned into TOPO-TA (Life Technologies). Cloned PCR products were Sanger sequenced using M13 forward and reverse primers to determine 3′ end junctions. Samples were sent for sequencing to Elim Biopharmaceuticals, Inc., Hayward, California. Lasergene 10 SeqBuilder software was utilized for sequence analysis. Flanking regions were mapped on the human reference genome hg19 (build 37) using Blat tool (https://genome.ucsc.edu/cgi-bin/hgBlat). The sequence data related to these insertions is included in Additional file 1: Table S3.

### Immunoblot analysis

To evaluate expression of CSA protein in the cells, HeLa, XPC-, XPD-, XPD+, CSA- and CSA+ cells were haverested in 300 μl of lysis buffer (50 mM Tris, pH 7.2, 150 mM NaCl, 0.5% Triton X-100, 10 mM EDTA, 0.5% SDS). After 10 min of sonication (Bioruptor, Diagenode, manufacturer’s recommended settings), lysates were clarified by centrifugation for 15 min at 4° at 13,000 rpm and the protein concentration was determined by Bradford assay (Biorad). 40 μg of protein was fractionated on a 4–12% bis-tris polyacrylamide gel (Life Technologies). Proteins were transferred to a nitrocellulose membrane using the iBlot gel transfer system from Life Technologies (manufacturer’s settings). The membrane was blocked for 1 h at room temperature in PBS (pH 7.4), 0.1% Tween 20 (Sigma), 5% skim milk powder (OXOID) and then incubated overnight at 4° with an anti-CSA monoclonal antibody (D-2, sc-376,981, Santa Cruz Biotechnology) diluted at 1:500 and an anti-GAPDH antibody (FL-335, sc-25,778, Santa Cruz Biotechnology) diluted at 1:1000 in PBS, 0.1% Tween 20, 3% non-fat dry milk. The membrane was then incubated for 1 h at room temperature with the secondary goat anti-mouse or donkey anti-rabbit HRP-conjugated antibody (sc-2005, sc-2313, Santa Cruz Biotechnology) diluted at 1:100,000 in PBS, 0.1% Tween 20, 3% non-fat milk. Signals were detected using Super Signal West Femto Chemiluminescent Substrate (Pierce).

### UV sensitivity assay

The protocol was adapted from [25]. Briefly 5 × 10^5^ cells were seeded in 6-cm plates and grown in growth medium for 24 h. The growth medium was removed and the cells were irradiated in the presence of 1 mL of 1X phosphate buffer saline (PBS) with a bactericidal UVC lamp (254 nm, 1.57 J/m^2^/s) at 0, 3, 6, 9 and 12 J/m^2^ UVC dose. The PBS was removed and replaced with growth medium. After 4 days, cells were counted with a hemocytometer to determine cell survival. Cell survival was calculated as the percent of live cells in the irradiated sample relative to the untreated sample.

### RNA-Seq analysis of HeLa gene expression

RNA was isolated from HeLa cells as described for RT-PCR. 5 μg of RNA was submitted to the University of Wisconsin Biotechnology Center (http://www.biotech.wisc.edu/services/dnaseq/services/Illumina) for polyA selection and strand-specific 2 × 100 bp RNA sequencing on an Illumina HiSeq2000. Approximately 40 million reads were subjected to RSEM analysis [26] on the human GR38 reference genome and output calculated for all of the ENCODE coding gene alignments in FPKM (fragments per kilobase per million reads).

## Results

### CSA protein does not control the rate of L1 retrotransposition

In GGR-deficient cells, we have observed an increase of 3–10-fold in L1 retrotransposition rate in comparison to the complemented cell lines, suggesting that the NER repair pathway limits L1 insertion to the genome [16]. We therefore wondered if the L1 retrotransposition rate would also increase in TCR-deficient cells. SV40-transformed, CSA-deficient (CSA-) skin fibroblasts were obtained from Coriell Cell Repository from a patient suffering from cockayne syndrome (see materials and methods). These cells express a truncated CSA mRNA that does not produce functional CSA protein and the cells are remarkably sensitive to UV light exposure ([22] and Additional file 2: Figure S1). We stably complemented the cells by transfection with a CSA cDNA expression vector under selection and controlled for the efficiency of the complementation with a functional UV sensitivity assay (Materials and Methods and [25]). The data revealed that the stably complemented (CSA+) cells are less sensitive to UV light exposure (Additional file 2: Figure S1A). RT-PCR and immunoblot assays confirmed the overexpression of CSA mRNA and protein in the stably complemented cells (Additional file 2: Figs. S1A and S1B).

To test the activity level of the L1 retrotransposon in CSA-deficient and complemented cells, we performed an L1 retrotransposition assay by transfecting the cells with the JM102/L1.3 vector expressing the L1.3 element tagged at the 3’end with *mneoI* retrotransposition cassette [19]. The retrotransposition cassette contains an antisense neomycin resistant gene, interrupted by a sense oriented intron that is spliced only in L1 mRNA (Fig. 2a). Therefore, the neo^R^ gene becomes expressed and functional only after retrotransposition. The assay allows for an estimation of L1 retrotransposition rate by counting Neo^R^ colonies 14 days after selection (Fig. 2b). In contrast to the results obtained in GGR-deficient cells, the retrotransposition assays do not show a rate increase in CSA- cells in comparison to isogenic CSA+ cells (Fig. 2c and Additional file 2: Figs. S2A-C). There were also no measurable differences in L1-caused toxicity in the cells or cell growth as shown in Fig. 2d and Additional file 2: Figs. S2D-F. This study suggests that if there is a difference of L1 retrotransposition rate in these cells, it is relatively minor, as we would have predicted based on the relatively small portion of the genome under surveillance by the TCR-NER pathway at any one time ([8] and see RNA-Seq gene expression data (Additional file 2: Figure S3)).

### *de novo* L1 inserts do not generate large duplications at the target site in CSA-deficient cells

**Fig. 3:**
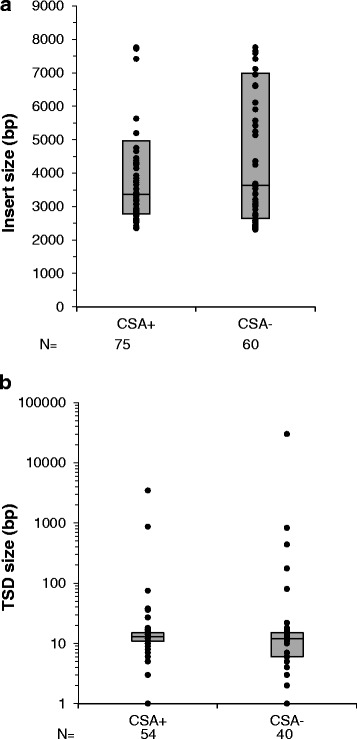
Characterization of L1 de novo insertions in CSA-deficient (CSA-) cells and in the stably complemented (CSA+) cell line. **a** and **b** Dot plot representation of the length of de novo L1 insertions in panel **a** and of the TSD size in panel **b** recovered from CSA-deficient cells (CSA-) and in CSA-complemented cells (CSA+). The boxes represent the interquartile range (between the first -bottom line- and the third –top line- quartiles) of the distribution of insertion size or TSD size for each cell line. The line in the middle of the box represents the median. The count (N) of recovered insertions for each cell line is indicated below the name of the cell line. The differences are not statistically significant (*P*-value = 1.049 for panel **a** *P*-value = 0.404 for panel **b** Mann-Whitney test)

In GGR-deficient cells, we also observed that abnormally large duplications (over 1 kb) were formed at the L1 insertion site [16]. We therefore decided to investigate the features of L1 *de novo* insertions in CSA-deficient and complemented cells (Additional file 1: Tables S1 and S2). We have recovered 60 and 75 L1 *de novo* insertions from CSA-deficient and complemented cells, respectively (Additional file 1: Tables S1 and S2), using the synL1_neo rescue vector and the previously published method (Materials and Methods section and [16, 20, 27]). Surprisingly, the characteristics of L1 *de novo* insertions were very similar in CSA- and CSA+ cells. No chromosome was specifically targeted by L1 *de novo* insertions. No significant difference was identified in the median length of the inserts in CSA+ and CSA- cells (3401 and 3642 bp respectively) (Fig. 3a). Additionally, we found about 21% of L1 *de novo* insertions were full length in both cells lines, consistent with 10% - 30% observed in previous studies [1, 4, 28–30]. Except for one recovered insert in CSA- cells, all L1 *de novo* insertions had a poly-A tail and their target site sequences were T-rich, close to the TTTT/A consensus sequence (Additional file 1: Tables S1 and S2; [4, 6, 20]). Deletions (2 to 2000 bp) at the target site of L1 *de novo* insertion were identified in 19 out of 60 insertions (31,6%) in CSA- deficient and in 21 out of 77 insertions (27%) in the complemented cells (Additional file 1: Tables S1 and S2). A high rate of genomic deletions was also reported in XPD+ and HeLa cells (47% and 26%, respectively) [4, 16]. Typical target-site duplications (TSDs) duplications were primarily observed at the target site of L1 *de novo* insertions recovered from CSA- and CSA+ cells (Additional file 1: Tables S1 and S2). The TSD size ranged from 1 to 29,902 bp in CSA- cells and from 1 to 3450 bp in CSA+ cells with a median length of 13 and 12 bp in CSA+ and CSA- cells, respectively (Fig. 3b). These data corresponded to the typical observations reported in HeLa cells or complemented NER cells (15 bp on average) [4, 16, 18] and were very different to the abnormally large TSDs (over 1 kb on average) observed in the other GGR-deficient cells [16].

### Does TCR-NER influence the insertional bias of L1 elements in genes?

We then investigated the distribution of L1 *de novo* insertions from our tagged vector in the genomes of CSA-deficient and complemented cells. Because the TCR pathway specifically excises the DNA lesion that interrupts the transcription process, it seems likely that a nascent L1 insert in the template strand would block transcription, and possibly trigger TCR to remove the inhibiting L1 retrotransposition event.

**Fig. 4:**
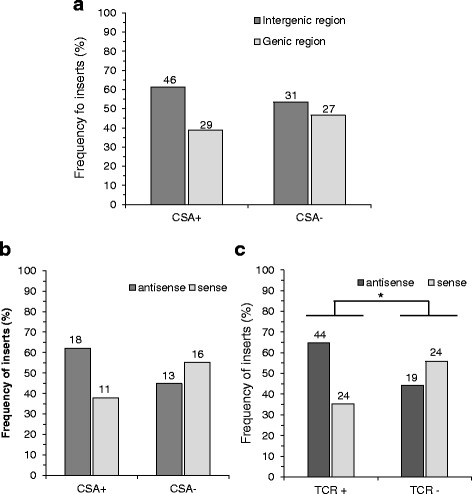
The tendency of L1 de novo insertions in the antisense orientation within genes decreased in the cells deficient in the TCR pathway (CSA- and XPD- cells). **a** Analysis of the distribution of L1 de novo insertions in the genome of CSA- and CSA+ cells. Bars represent the frequency of de novo L1 insertions in genic and intergenic regions of the genome. The numbers over the bars represent the counts of recovered insertions for each condition. The difference in the genomic repartition of the insertions between CSA+ and CSA- cells is not statistically significant (*p* = 0.227; Fisher exact test). **b** Analysis of the orientation of L1 de novo insertions within genes in CSA+ and CSA- cells. Bars represent the frequency of de novo L1 insertions in sense and antisense orientation within genes. The numbers over the bars represent the counts of recovered insertions for each condition. The difference in the counts of sense-oriented vs antisense-oriented recovered L1 insertions between CSA+ and CSA- cells is not statistically significant (*p* = 0.292; Fisher exact test). **c** Analysis of the orientation of L1 de novo insertions within genes in TCR proficient (TCR+: HeLa, CSA+, XPD+, XPC-) and deficient (TCR-: XPD-, CSA-) cell lines. Bars represent the frequency of de novo L1 inserts in sense and antisense orientation within genes in TCR+ and TCR- cell lines. The numbers over the bars represent the combinations of the counts of recovered L1 insertions in HeLa, CSA+, XPD+ and XPC- cells (TRC+ cell lines) and in XPD- and CSA- cells (TRC- cell lines). The difference in the counts of sense-oriented vs antisense-oriented recovered L1 insertions between TCR+ and TCR- cell lines is statistically significant (*p* = 0.049; Fisher exact test)

As observed in the reference genome and in many cell lines (Additional file 2: Figure S4A and [3]), L1 *de novo* insertions were almost equally dispersed in genic and intergenic regions of the genome of both CSA- and CSA+ cells (Additional file 1: Tables S1 and S2 and Fig. 4a). Nevertheless, when L1 *de novo* insertions were integrated within genes in the complemented CSA+ cells, we characterized twice as many antisense-oriented as sense-oriented insertions (62.1% and 37.9% respectively) (Fig. 4b). This observation agreed with the previously reported trends for the genomic orientation of L1 elements in genes [4, 21, 31] (see Additional file 2: Figure S3), L1 *de novo* insertions in HeLa cells (see Additional file 1: Table S4) and brain cells [4, 7]. In contrast, L1 *de novo* insertions showed no significant bias in sense versus antisense orientation in CSA-deficient cells (Fig. 4b).

We reasoned that if the TCR sub-pathway would influence L1 orientation in genes, any steps in the pathway downstream from the sensor (CSA) would influence similarly L1 *de novo* insertions, while the sensor for the GGR sub-pathway (XPC) would not have the same effect. XPC-deficient cells showed a similar orientation bias for L1 *de novo* insertions to those seen in other TCR-proficient cells (Additional file 2: Figure S4B). However, L1 *de novo* insertions were equally sense and antisense oriented in XPD-deficient cells, which are defective for the downstream NER pathway factor that affects both TCR and GGR (Additional file 2: Figure S4B). In XPD+ cells, the complemented version of XPD- cells, the orientation bias was again observed for L1 *de novo* insertions (Additional file 2: Figure S4B).

In conclusion, our results revealed that L1 *de novo* insertions were preferentially antisense oriented in cells proficient for the TCR pathway (TCR+, Fig. 4c), such as HeLa, CSA+, XPD+ cells as well as XPC- cells. In TCR-deficient cells (TCR-, Fig. 4c), such as CSA- and XPD- cells, the orientation of L1 *de novo* insertions within genes was random (Fig. 4c).

### Expression of genes in which L1 inserted in HeLa cells

Because TCR is only active when a transcription complex hits a DNA lesion on the template strand, we predicted that sense-strand L1 insertions (occurring in the template strand) would be depleted in actively transcribing genes relative to the antisense-oriented insertions that would not be predicted to be affected by TCR (see Additional file 2: Figure S5). We therefore carried out a quantitation of gene expression for the ENCODE coding sequences in the human genome from HeLa cells. HeLa cells were chosen because they have an intact TCR pathway and because there is more available data on *de novo* inserts in HeLa cells than any other cell line.

In this study, approximately 80% of the cellular genes had little or no transcription (Additional file 2: Figure S3) confirming that they would be unlikely targets for TCR. Many of the expressed genes had expression levels less than 1 % the level of GAPDH, suggesting that they might be less subject to TCR than more actively transcribed genes.

When we examined HeLa *de novo* inserts analyzed with the rescue approach utilized in this manuscript from Gilbert et al. [4] and from this study, we see 39 inserts in the antisense orientation relative to ENCODE genes and 17 in the sense orientation (Additional file 1: Table S1). This ratio of antisense to sense is very similar to the ratio seen in the genome [3]. When we look at the expression levels from those genes, we see that the genes with antisense inserts have an average FPKM expression value of almost 25, while the sense inserts are in genes with less than 10 FPKM. This is significant at the 0.04 level in a two-tailed T-test. Furthermore, given that the majority of ENCODE genes have no measured expression, it is interesting that even though the genes in which the insertions occurred are not highly expressed, there is also a depletion of insertions in non-expressed genes. We are not sure if this represents a preferred target for insertion or the requirement for open chromatin to allow the selectable marker in the L1 element to express.

## Discussion

Although L1 retrotransposons are inserted throughout the human genome, these autonomous mobile elements have been found to be located with a strong antisense bias within genes [3]. This orientation bias is a characteristic of referenced and established L1 elements as well as polymorphic and *de novo* insertions (Additional file 1: Table S1) [4, 7]. Although it has been suggested that the bias may be the result of selection eliminating the insertions in the sense orientation that might be more disruptive of gene expression [32], this seems unlikely to have a strong influence on the *de novo* insertions. Thus, it is worth considering whether there is a specific mechanism limiting sense insertion in genes, possibly limiting the mutagenic impact of these insertion events. In the present study, we have demonstrated that recovered L1 *de novo* insertions are equally sense and antisense oriented within active genes in CSA- and XPD- deficient cells, both defective in the TCR pathway. These results suggest that the TCR pathway is responsible for much of the orientation bias of L1 elements in the human genome, although we cannot rule out some post-insertional selection influences as well. This demonstrates that in addition to the influence of GGR on L1 retrotransposition rate, the TCR subpathway also influences the distribution of inserts.

**Fig. 5:**
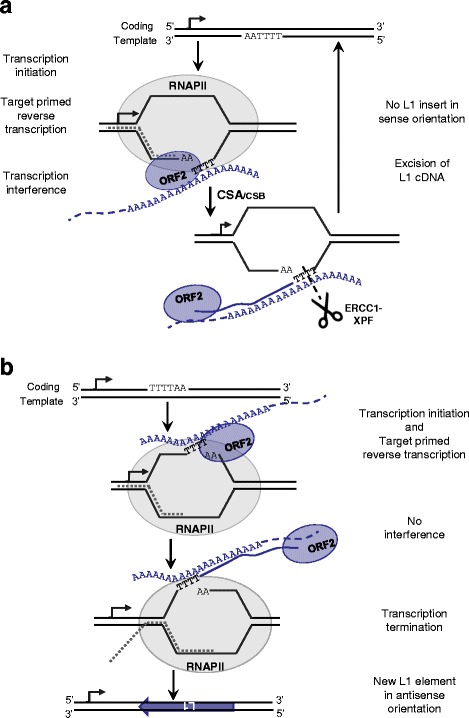
The TCR pathway prevents the L1 retrotransposon from inserting into the template strand during the transcription of active genes. **a** If an L1 insertion targets the template strand during the transcription of the gene, it can interfere with the RNAPII complex and stop transcription. CSA and CSB proteins detect the stalled RNAPII and recruit the other proteins of the NER pathway. The elongating L1 cDNA is then cleaved and the original DNA sequence is restored allowing completion of the gene transcription. This process results in the depletion of sense L1 insertions in actively transcribed genes. **b** If the L1 machinery targets the coding strand during the gene transcription, it does not interfere with the RNAPII complex. The transcription as well as the L1 insertion process can be completed. Thus, a de novo L1 insert ends up in antisense orientation within the active gene

In cells proficient for TCR, the pathway is recruited at stalled RNAPII complex and excises DNA lesions blocking the RNAPII processivity on the template strand (Fig. 1). After the repair, the transcription process is re-initiated. If L1 elements insert in the template strand of a gene, they would end up in the same orientation as the gene [33]. Insertions in the coding strand that would result in antisense insertions would not be expected to stall RNAPII and induce TCR (Fig. 5a). The data presented in our study suggest that the TCR pathway may prevent the insertion of L1 elements in the template strand of actively transcribed genes, but not in the coding strand, leading to the observed orientation bias of L1 inserts in the genome. This is supported by both the ratios of sense to antisense inserts (Fig. 4), as well as the tendency for sense inserts to be present in less expressed genes (Additional file 1: Table S1) than antisense inserts in HeLa cells with active TCR. Conversely, if the L1 machinery targets the coding strand, there would be no interference with the RNAPII complex and a L1 *de novo* insertion would be able to occur (Fig. 5b). The *de novo* insertion would be in antisense orientation within the gene.

Our data are consistent with the model that the TCR pathway may minimize interference of gene expression by new L1 retrotransposition events. We did not observe a strong effect of the TCR regulation on the overall L1 retrotransposition rate because only a small part of the genome is actively and efficiently transcribed at any given time in a cell (see Additional file 2: Figure S3) and the rest can still be protected from *de novo* L1 insertion by the GGR pathway (Additional file 2: Figure S5). The TCR pathway, which is essential for the protection of gene expression, represents a unique mechanism in the regulation of L1 retrotransposition especially during embryonic development when L1 activity is high [34] and L1-caused mutations could be detrimental for cell survival.

Although L1 elements are distributed throughout the genome, there are likely to be multiple factors that influence their distribution. L1 elements preferentially insert into a locally A + T-rich target sequence [6, 35, 36]. Thus, it is likely that the relative density of such A + T-rich target sequences may influence the rate of insertion in those regions. In addition, insertion of L1 sequences into genes may provide various signals that either fully or partially disrupt expression of the gene [37–39] resulting in negative selective pressure that will eventually lead to depletion of genes in which L1 insertions have occurred [40–43] This is likely a contributor to the relative paucity of L1 within genes that increases over evolutionary time [3, 41]. The insertion of L1 sequences may be more disruptive in one orientation relative to another [3] which could also lead to selection for more L1 elements in one orientation relative to another within genes. However, our finding that TCR can contribute strongly to such an insertion bias provides a mechanism that may establish such a bias immediately, without requiring time for selective pressure to alter the frequency.

## Conclusions

This work shows that the previously observed bias against sense-oriented L1 elements in genes is primarily due to transcription-coupled nucleotide excision repair being able to block sense insertions, rather than principally being due to selection post insertion. This would serve to minimize the negative impact of L1 insertions on gene expression.

## Additional files

Additional file 1: Table S1. Characteristics of recovered *de novo* L1 inserts in CSA-deficient cells. This table describes the general characteristics of the L1 inserts isolated from the CSA-minus cells. **Table S2.** Characteristics of recovered *de novo* L1 inserts in stably complemented CSA + cells. This table describes the general characteristics of the L1 inserts isolated from the cells that have been complemented to be CSA+. **Table S3A&B.** DNA sequences flanking rescued L1 inserts. S3A has the sequence data from the L1 insertion rescues for the CSA-minus cells, while S3B has similar data for the complemented cells that are now CSA plus. **Table S4.** FPKM values for *de novo* L1 inserts in HeLa cells that inserted within genes. (ZIP 130 kb)

Additional file 2: Figure S1. Control for the efficiency of the complementation of CSA-deficient cells. **Figure S2.** L1 retrotransposition rate is not significantly different in CSA-deficient cells (CSA-) and in the stably complemented CSA-deficient cells (CSA+). **Figure S3.** FPKM counts for Encode genes expressed in HeLa. **Figure S4.** The tendency of *de novo* L1 elements to insert in the antisense orientation within genes is lost in the cells deficient in the TCR pathway (CSA- and XPD- cells). **Figure S5.** Model of regulation of L1 insertion in genes by the TCR pathway. (ZIP 241 kb)

## Abbreviations

bp: Base pairs
CMV: Cytomegalovirus
CSA: Cockayne syndrome protein A
CSB: Cockayne syndrome protein B
eGFP: Enhanced green fluorescent protein
ERCC1: Excision repair 1
FPKM: Fragments per kilobase per million reads
GGR: Global genome repair sub-pathway of NER
IRES: Internal ribosome entry site
J/m^2^/s: Joules per meter squared per second
L1: LINE-1 or Long, INterspersed Element-1
mblast: Blasticidin resistance cassette
mneo: Neomycin resistance cassette
Neo^R^: Neomycin or geneticin resistance
NER: Nucleotide excision repair
nt: Nucleotide
PBS: Phosphate-buffered saline
PCR: Polymerase chain reaction
RNAPII: RNA polymerase II
RNA-Seq: Next generation sequencing protocol for RNA
RT-PCR: Reverse transcription – PCR
SV40: Simian virus 40
TCR: Transcription-coupled repair
TSD: Target site duplication
UV: Ultraviolet
XPA: Xeroderma pigmentosum protein A
XPC: Xeroderma pigmentosum protein C
XPD: Xeroderma pigmentosum protein D
XPF: Xeroderma pigmentosum protein F
