# Supplementary material for: Transcription coupled repair and biased insertion of human retrotransposon L1 in transcribed genes
Source: Mob DNA. 2017 Dec 6;8:18. doi: 10.1186/s13100-017-0100-5 (PMC5717806; doi:10.1186/s13100-017-0100-5)
Supplement: Supplementary file 1 — Characteristics of recovered de novo L1 inserts in CSA-deficient cells. This table describes the general characteristics of the L1 inserts isolated from the CSA-minus cells. Table S2. Characteristics of recovered de novo L1 inserts in stably complemented CSA + cells. This table describes the general characteristics of the L1 inserts isolated from the cells that have been complemented to be CSA+. Table S3A&B. DNA sequences flanking rescued L1 inserts. S3A has the sequence data from the L1 insertion rescues for the CSA-minus cells, while S3B has similar data for the complemented cells that are now CSA plus. Table S4. FPKM values for de novo L1 inserts in HeLa cells that inserted within genes. (ZIP 130 kb) [file 13100_2017_100_MOESM1_ESM.zip › Supplementaal Table S4.docx]

**Supplemental TABLE S4. FPKM values for de novo L1 inserts in HeLa cells that inserted within genes**.

The data listed as ‘clone #’ come from Gilbert et al. [4]

The data listed as HB.# or HZ.# come from this study (Supplementary Table 3)
