# Supplementary material for: Transcription coupled repair and biased insertion of human retrotransposon L1 in transcribed genes
Source: Mob DNA. 2017 Dec 6;8:18. doi: 10.1186/s13100-017-0100-5 (PMC5717806; doi:10.1186/s13100-017-0100-5)
Supplement: Supplementary file 2 — Control for the efficiency of the complementation of CSA-deficient cells. Figure S2. L1 retrotransposition rate is not significantly different in CSA-deficient cells (CSA-) and in the stably complemented CSA-deficient cells (CSA+). Figure S3. FPKM counts for Encode genes expressed in HeLa. Figure S4. The tendency of de novo L1 elements to insert in the antisense orientation within genes is lost in the cells deficient in the TCR pathway (CSA- and XPD- cells). Figure S5. Model of regulation of L1 insertion in genes by the TCR pathway. (ZIP 241 kb) [file 13100_2017_100_MOESM2_ESM.zip › 13100_2017_100_MOESM2_ESM/Supplemental figure S1.pdf]

Supplemental Figure S1

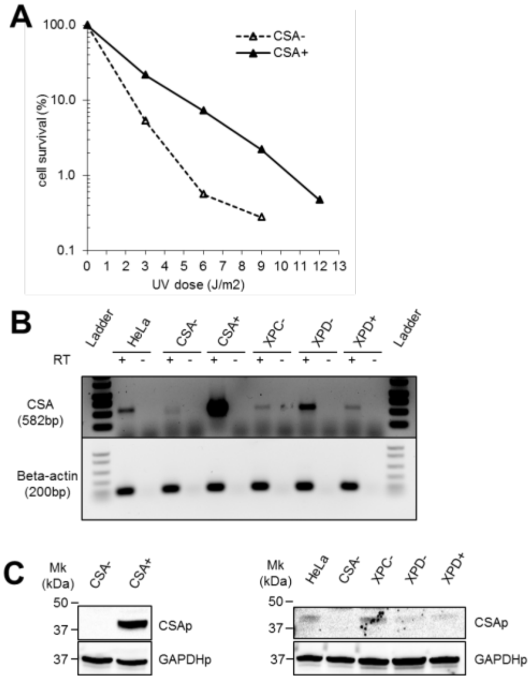

Figure S1. Control for the efficiency of the complementation of CSA-deficient cells.

A. Stably complemented CSA-deficient cells (CSA+) are more resistant to UV light exposure. The UV sensitivity assay is a functional assay to control for the NER deficiency, as NER-deficient cells are very sensitive to UV light exposure. CSA+ and CSA- cells were plated in 10-cm dishes and exposed the next day to 0, 3, 6, 9, and 12 J/m2 UV dose. The number of cells surviving the treatment was determined 4 days after exposure. The graph represents the logarithm of the cell survival percentage plotted against the UV dose (J/m2).

B. Analysis of CSA mRNA synthesis in HeLa, CSA-, CSA+, XPC-, XPD-, XPD+ cells. 1ug of whole-cell mRNA was reverse transcribed using poly dT primers at 42C for 1 hour. PCR amplification of the 5' end of the CSA cDNA was then performed to determine the presence of the CSA mRNA. Beta-actin was used as a control. PCR products were run on a 1% agarose gel containing ethidium bromide. The CSA PCR product was detected in every cell line, with different intensity, reflecting the amount of the RNA in the cells. In CSA- cells, none of CSA coding sequence is intact: allele 1 has a substitution C-to-T at nt37 introducing a stop codon early in the protein and allele 2 contains a substitution G-to-T at nt479 transforming an alanine in a valine in the middle of the protein (Ridley, 2005, J hum genet). We purified and sequenced the PCR products in CSA- and CSA+ cells. We were able to detect the mutation in allele 1, but not the mutation in the second allele because of a deletion of 83 nucleotides in the recovered cDNA (data not shown). Therefore, the PCR amplification in CSA- cells is faint and smeary. In the other cell lines, the PCR product is a very distinct band and matches with the sequence of CSA cDNA.

C. Analysis of CSA protein (CSAp) expression in CSA-, CSA+, HeLa, XPC-, XPD-, XPD+ cells. 40ug of cellular extracts were loaded on 4-12% polyacrylamide gel (Life Technology) to detect CSA protein (44kDa). Western blot assays were performed using monoclonal anti-CSA antibody (Santa Cruz Biotechnology). Polyclonal anti-GAPDH antibody (Santa Cruz Biotechnology) was used as a loading control. CSA protein expression is very low in HeLa, XPC-, XPD-, and XPD+ cell lines. CSA protein is not expressed in CSA- cells. Overexpression of CSA protein is detected in CSA+ cells.
