# Supplementary material for: Transcription coupled repair and biased insertion of human retrotransposon L1 in transcribed genes
Source: Mob DNA. 2017 Dec 6;8:18. doi: 10.1186/s13100-017-0100-5 (PMC5717806; doi:10.1186/s13100-017-0100-5)
Supplement: Supplementary file 2 — Control for the efficiency of the complementation of CSA-deficient cells. Figure S2. L1 retrotransposition rate is not significantly different in CSA-deficient cells (CSA-) and in the stably complemented CSA-deficient cells (CSA+). Figure S3. FPKM counts for Encode genes expressed in HeLa. Figure S4. The tendency of de novo L1 elements to insert in the antisense orientation within genes is lost in the cells deficient in the TCR pathway (CSA- and XPD- cells). Figure S5. Model of regulation of L1 insertion in genes by the TCR pathway. (ZIP 241 kb) [file 13100_2017_100_MOESM2_ESM.zip › 13100_2017_100_MOESM2_ESM/Supplemental Figure S4.docx]

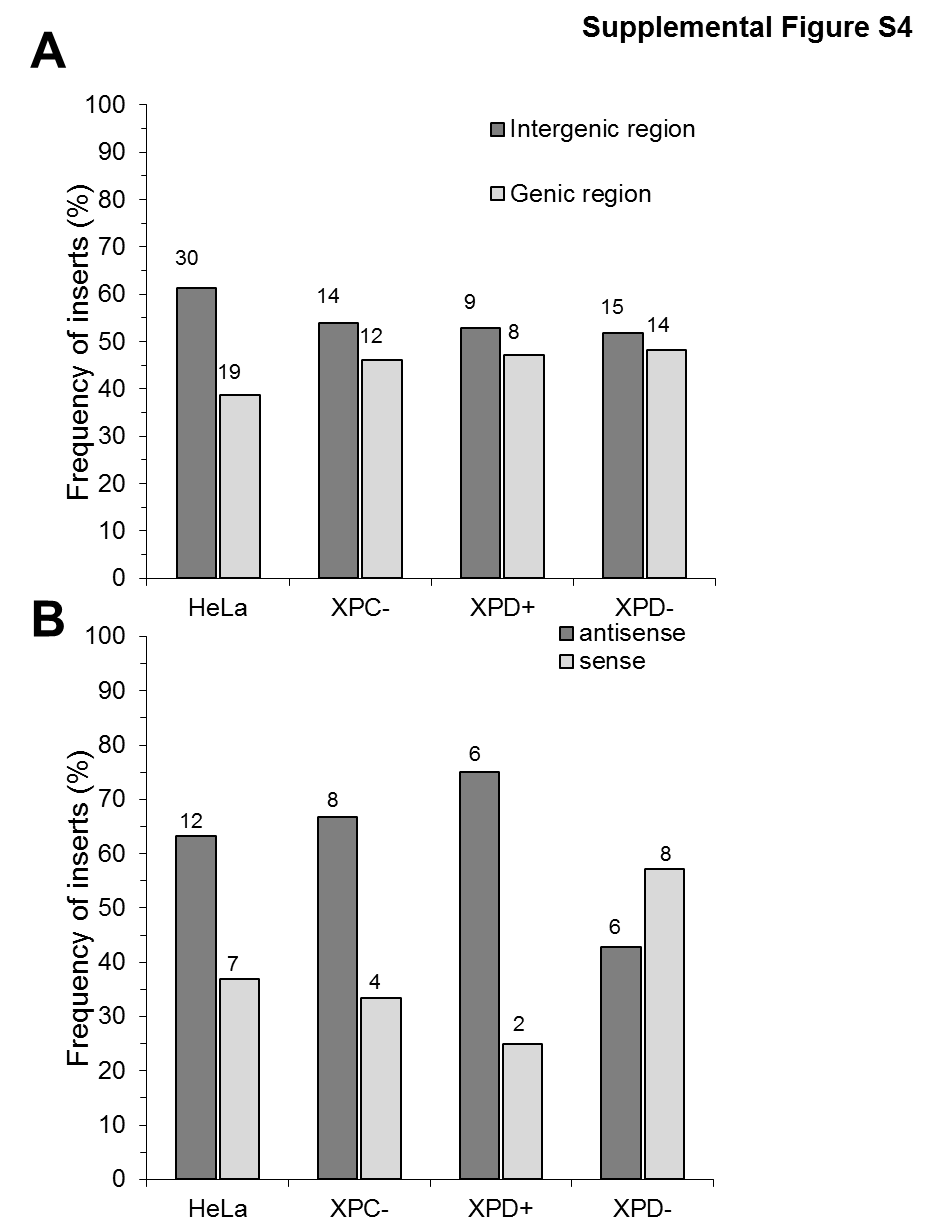


**Supplemental Figure S4.** The tendency of d*e novo* L1 elements to insert in the antisense orientation within genes is lost in the cells deficient in the TCR pathway (CSA- and XPD- cells).

**A.** Analysis of the distribution of L1 new inserts recovered from HeLa, XPC-, XPD+ and XPD- cells. Bars represent the frequency of *de novo* L1 inserts in genic and intergenic regions of the genome. The numbers over the bars represent the counts of recovered inserts for each condition.

**B.** Analysis of the orientation of L1 new elements within genes in HeLa, XPC-, XPD+ and XPD- cells. Bars represent the frequency of *de novo* L1 inserts in sense and antisense orientation within genes. The numbers over the bars represent the counts of recovered inserts for each condition.
