# Supplementary material for: Transcription coupled repair and biased insertion of human retrotransposon L1 in transcribed genes
Source: Mob DNA. 2017 Dec 6;8:18. doi: 10.1186/s13100-017-0100-5 (PMC5717806; doi:10.1186/s13100-017-0100-5)
Supplement: Supplementary file 2 — Control for the efficiency of the complementation of CSA-deficient cells. Figure S2. L1 retrotransposition rate is not significantly different in CSA-deficient cells (CSA-) and in the stably complemented CSA-deficient cells (CSA+). Figure S3. FPKM counts for Encode genes expressed in HeLa. Figure S4. The tendency of de novo L1 elements to insert in the antisense orientation within genes is lost in the cells deficient in the TCR pathway (CSA- and XPD- cells). Figure S5. Model of regulation of L1 insertion in genes by the TCR pathway. (ZIP 241 kb) [file 13100_2017_100_MOESM2_ESM.zip › 13100_2017_100_MOESM2_ESM/Supplemental Figure S5.docx]

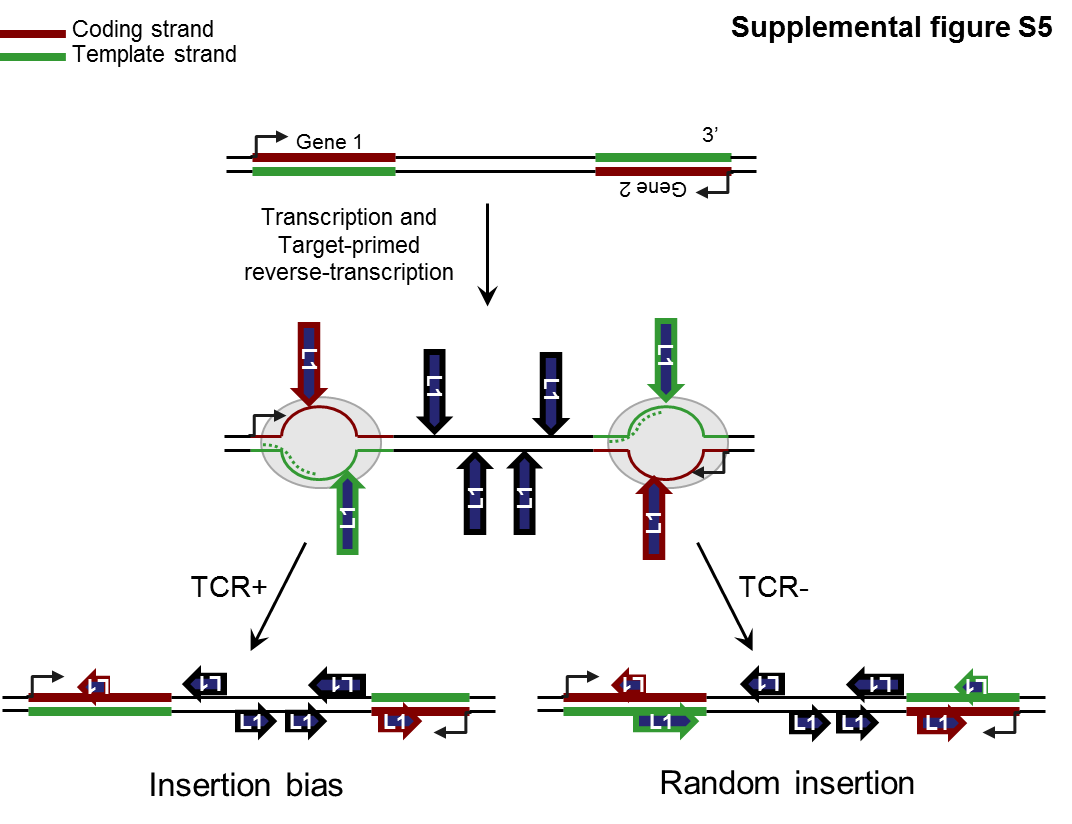


**Supplemental Figure S5.** Model of regulation of L1 insertion in genes by the TCR pathway.

The TCR pathway limits L1 insertion targeting the template strand (green line) of active genes. L1 machinery can target the coding strand (red line) without interference with the RNAPII complex. Therefore, L1 elements are mainly inserted in the antisense orientation (red arrows) within genes and the proportion of L1 elements in the sense orientation (green arrows) is low. If the TCR pathway is not functional, the L1 insertion process targeting the template strand is not interrupted and can be completed. Therefore, the proportion of L1 elements in sense and antisense orientations within genes is almost equal. L1 machinery targeting the intergenic region (black line) of the genome, not actively transcribed, is not regulated by the TCR pathway. L1 elements in intergenic region (black arrow) are present in both strands indifferently.
